# Men’s perspectives of prostate cancer screening: A systematic review of qualitative studies

**DOI:** 10.1371/journal.pone.0188258

**Published:** 2017-11-28

**Authors:** Laura J. James, Germaine Wong, Jonathan C. Craig, Camilla S. Hanson, Angela Ju, Kirsten Howard, Tim Usherwood, Howard Lau, Allison Tong

**Affiliations:** 1 Sydney School of Public Health, The University of Sydney, Sydney, Australia; 2 Centre for Kidney Research, The Children’s Hospital at Westmead, Westmead, Australia; 3 Centre for Transplant and Renal Research, Westmead Hospital, Westmead, Australia; 4 Department of General Practice, Westmead Clinical School, The University of Sydney, Sydney, Australia; 5 The George Institute for Global Health, Sydney, Australia; 6 Department of Urology, Westmead Hospital, Westmead, Australia; UCL, UNITED KINGDOM

## Abstract

**Background:**

Prostate cancer is the most commonly diagnosed non-skin cancer in men. Screening for prostate cancer is widely accepted; however concerns regarding the harms outweighing the benefits of screening exist. Although patient’s play a pivotal role in the decision making process, men may not be aware of the controversies regarding prostate cancer screening. Therefore we aimed to describe men’s attitudes, beliefs and experiences of prostate cancer screening.

**Methods:**

Systematic review and thematic synthesis of qualitative studies on men’s perspectives of prostate cancer screening. Electronic databases and reference lists were searched to October 2016.

**Findings:**

Sixty studies involving 3,029 men aged from 18–89 years, who had been screened for prostate cancer by Prostate Specific Antigen (PSA) or Digital Rectal Examination (DRE) and not screened, across eight countries were included. Five themes were identified: Social prompting (trusting professional opinion, motivation from family and friends, proximity and prominence of cancer); gaining decisional confidence (overcoming fears, survival imperative, peace of mind, mental preparation, prioritising wellbeing); preserving masculinity (bodily invasion, losing sexuality, threatening manhood, medical avoidance); avoiding the unknown and uncertainties (taboo of cancer-related death, lacking tangible cause, physiological and symptomatic obscurity, ambiguity of the procedure, confusing controversies); and prohibitive costs.

**Conclusions:**

Men are willing to participate in prostate cancer screening to prevent cancer and gain reassurance about their health, particularly when supported or prompted by their social networks or healthcare providers. However, to do so they needed to mentally overcome fears of losing their masculinity and accept the intrusiveness of screening, the ambiguities about the necessity and the potential for substantial costs. Addressing the concerns and priorities of men may facilitate informed decisions about prostate cancer screening and improve patient satisfaction and outcomes.

## Introduction

Prostate cancer is the most commonly diagnosed non-skin cancer in men and accounts for 48 deaths per 100,000 men per year in the UK.[1] One in seven men in Australia and the UK will develop prostate cancer by age 75 years.[2, 3] The overall age-standardised incidence is 182 per 100,000 men per year in the UK,[4] and 163 and 129 per 100,000 men per year in Australia and US respectively.[5, 6]

Screening for prostate cancer using prostate-specific antigen (PSA) is widely used in the general population, contributing to a threefold increase in the incidence of diagnosed prostate cancer.[7] However, screening remains controversial, in part due to conflicting results from recent large randomised controlled trials. One large-scale trial, conducted in the US, found no difference in prostate cancer mortality between the screened and control group after 13 years,[8] whereas another European trial, with a similar follow up, demonstrated a 21% relative reduction in the risk of prostate cancer-specific death.[9]

More recently, concerns about overdiagnosis have also been raised.[10–12] Early detection through screening can lead to overdiagnosis, which consequently may require further unnecessary investigations, including prostatectomy and radiation therapy.[10] It remains uncertain whether screening confers appreciable survival benefits, and treatment of screen detected disease can lead to adverse events including urinary incontinence, erectile dysfunction, bowel problems, and unwarranted anxiety.[10, 13] As a result, current guidelines for PSA testing vary worldwide.

The conflicting trial results, potential harms associated with the screening algorithm, and the inconsistent recommendations regarding PSA testing in clinical practice guidelines,[2, 11, 14–16] highlights the critical role of patient preference. Shared informed decision making is widely advocated to ensure men can understand and weigh the benefits and harms of prostate cancer screening.[2, 11, 12, 14, 16–18] However, men may not be aware of the potential risks of treating screen-detected prostate cancer.[19–21] Although a clinician’s recommendation to screen is associated with higher uptake of prostate cancer screening[22–25], embarrassment,[26–29] perceived low risk,[28, 30] and fear of cancer [28, 31, 32] explain men’s decisions not to undergo screening. We aimed to describe the beliefs, attitudes and experiences of men on prostate cancer screening. A broader range of insights across populations and healthcare settings will provide a comprehensive understanding of men’s perspectives on prostate cancer screening, and may also enable comparisons across demographic characteristics. This may inform strategies for informed, shared-decision making that explicitly considers their values and preferences.

## Methods

This study follows the Enhancing Transparency of Reporting the Synthesis of Qualitative research (ENTREQ) framework.[33] ENTREQ covers five main domains for reporting the systematic review of primary qualitative studies: introduction, methods and methodology, literature search and selection, appraisal, and synthesis of findings.

### Selection criteria

We included qualitative studies that used interviews or focus groups which elicited perspectives of adult men, of any age, on prostate cancer screening. To obtain a broad range of perspectives on attitudes to screening, studies of men with no previous history of screening or had been screened for prostate cancer using PSA or digital rectal examination (DRE) were included. We excluded studies that only included men diagnosed with prostate cancer, or that included health professionals. Studies published in non-English language were excluded to prevent cultural and linguistic bias in translations. We also excluded studies if they used structured surveys and reported only quantitative data, or were epidemiological studies, editorials or reviews.

### Data sources and searches

We searched MEDLINE, Embase, PsycINFO and Cumulative Index to Nursing and Allied Health Literature (CINAHL) from inception to October 2016 (S1 Text), as well as Google Scholar and the reference lists of relevant studies and reviews. Author (LJJ) screened titles and abstracts of search results and excluded those that did not meet the selection criteria. Full texts of the remaining articles were assessed for study eligibility by LJJ.

### Quality of reporting assessment

Three authors (LJJ/CSH/AJ) independently evaluated the transparency of reporting of each included study using the Consolidated Criteria for Reporting Qualitative Research (COREQ) framework.[34] The COREQ items addresses the research team, relationship with participants, participant selection, setting, data collection, data analysis and reporting. Discrepancies were discussed and resolved among the reviewers.

### Synthesis

We synthesised the results from all included studies using thematic synthesis, as described by Thomas and Harden.[35] All text and quotations included within the “Results” or “Discussion/Conclusion” section from the included studies were imported into HyperRESEARCH (ResearchWare, INC. 2015, version 3.7.1) software for qualitative data management. LJJ read each study, conducted line-by-line coding of the findings, and inductively identified concepts relating to prostate cancer screening perspectives and grouped similar concepts into themes and subthemes. Three authors (LJJ, CSH, AJ) read the papers independently to ensure that the range and depth of data reported in the included studies were included in the analytical framework. Conceptual links among these themes were identified and mapped into a thematic schema.

## Results

### Literature search

**Fig 1:**
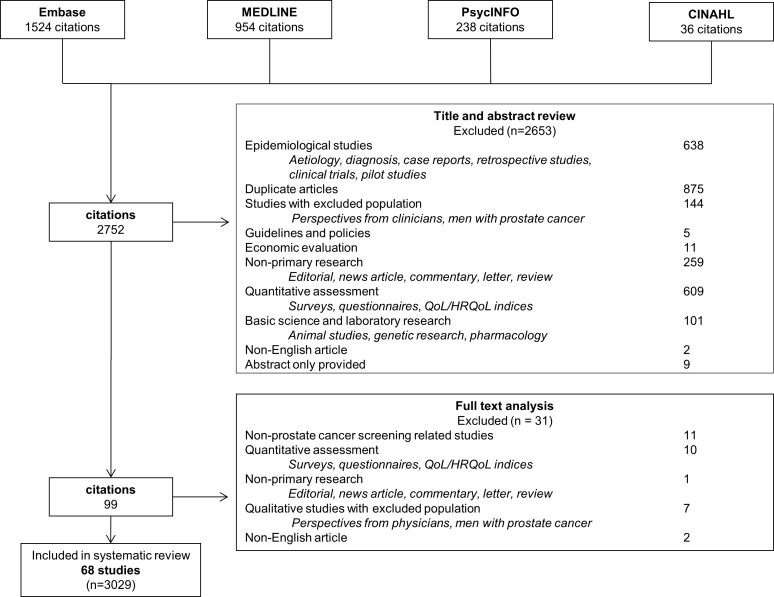
Search process and results.

Of the 2,752 articles retrieved, 68 eligible studies involving 3,029 participants were identified (Fig 1). The characteristics of the studies are provided in Table 1 and S1 Table. Forty studies (59%) included men with and without a history of screening. Nine (13%) studies included men who had not been screened, and 4 (6%) studies included men who had been previously screened. Fifteen (22%) studies did not report participant’s history of screening. Both methods for screening, PSA or DRE, were used, however the number of men that used either method was not reported in most studies. The studies were conducted in nine countries, including the United States (49 [72%]; of which 26 (38%) only included African American men), United Kingdom (7 [10%]), Australia (6 [9%]), Barbados, Belgium, Canada, Germany, Nigeria and Uganda (1 study each).

**Table 1:** Characteristics of included studies (n = 68).

Study characteristics	No. of studies	n (%)
Year of publication		
1996–2000	5	7
2001–2005	17	25
2006–2010	21	31
2011–2016	25	37
Country		
United States	49	72
United Kingdom	7	10
Australia	6	9
Other*	6	9
Sample size**		
1 to 20	22	32
21 to 40	19	28
41 to 60	10	15
61 to 80	9	13
81–100	4	6
>100	3	4
Not reported	1	2
Screening history		
Screened	4	6
Not screened	9	13
Both screened/not screened	40	59
Not reported	15	22
Method of data collection		
Focus groups	34	50
Interviews	27	40
Interviews and focus groups	7	10

*Barbados, Belgium, Canada, Germany, Nigeria, Uganda (1 study each)

** Only men

### Comprehensiveness of reporting of included studies

The comprehensiveness of reporting was variable, with studies reporting 7 to 23 of the 25 items in the adapted COREQ checklist (Table 2). The participant selection strategy was described in 58 (85%) studies, and data saturation was reported in 28 (41%) studies. The use of software to facilitate data analysis was reported in 41 (60%) studies, and 52 (76%) reported on the use of researcher triangulation. Almost all studies (66 [97%]) provided participant quotations.

**Table 2:** Comprehensiveness of reporting in included studies.

Reporting Criteria	No (%) N = 68		References of studies reporting each criterion
**Characteristics of research team:**			
Interviewer or facilitator identified	27	(40)	[19, 27, 36–56] [57–60]
Experience and training	30	(44)	[19–21, 37, 38, 42, 46, 47, 49, 55, 61–76] [57–60]
**Relationship with participants:**			
Relationship established before study started	9	(13)	[42, 45, 61, 68, 70, 77, 78] [57, 60]
**Participant Selection:**			
Sampling method *(e*.*g*. *snowball*, *purposive*, *convenience)*	58	(85)	[19–21, 27, 28, 36–46, 48–50, 52–56, 62–69, 71, 73, 75–90] [57–60, 91–94]
Method of approach	65	(96)	[19–21, 27, 28, 36–49, 51–56, 61–75, 77–90, 95–97] [57–60, 91–94]
Sample size	68	(100)	[19–21, 27, 28, 36–56, 61–90, 95–98] [57–60, 91–94]
Number or reasons for non-participation	19	(28)	[19, 20, 36–39, 43, 44, 48, 49, 52, 53, 56, 75, 79, 83] [57, 59, 60]
**Setting:**			
Setting of data collection	45	(66)	[20, 27, 36–47, 52–56, 62, 65, 66, 68–70, 73–75, 78, 82–84, 88–90, 96–98] [57–60, 91–93]
Presence of non-participants	3	(4)	[59, 60, 94]
Description of sample	66	(97)	[19–21, 27, 28, 36, 38–56, 61, 62, 64–90, 95–98] [57–60, 91–94]
**Data Collection:**			
Interview guide	58	(85)	[19–21, 27, 28, 36–46, 49, 50, 52–54, 56, 61–71, 73–78, 80–84, 87–89, 96–98] [57–60, 91–94]
Repeat interviews	36	(53)	[20, 21, 27, 28, 37–39, 41, 42, 46, 49–51, 54, 55, 61, 64, 66–69, 71–74, 77, 80–82, 84, 87, 90, 96, 97] [57, 60]
Audio or visual recording	63	(93)	[19–21, 27, 28, 36–55, 61–68, 70–82, 84, 85, 87–90, 95–98] [57–60, 91–93]
Field notes	31	(46)	[19–21, 27, 37, 39–43, 45, 47, 62–65, 67, 68, 71, 76, 78, 80, 82, 85, 87, 88, 90] [57, 58, 60, 91]
Duration	48	(71)	[19–21, 27, 28, 36–41, 43–46, 48, 51, 53–55, 62, 64–67, 69–71, 73, 74, 77–80, 82, 84, 86, 88, 90, 96, 97] [57–60, 91–93]
Translation and interpretation	6	(9)	[37, 53, 56, 68, 72, 84]
Protocol for data preparation and transcription	63	(93)	[19–21, 27, 28, 36–38, 40–42, 44–55, 61–75, 77–80, 82–90, 95–98] [57–60, 91–94]
Data (or theoretical) saturation	28	(41)	[36, 37, 40, 44–48, 53, 54, 62, 63, 65, 66, 70, 72, 77–79, 82, 83, 85, 86] [57, 58, 60, 91, 93]
**Data Analysis:**			
Researcher/expert triangulation (multiple researchers involved in coding and analysis)	52	(76)	[19, 21, 27, 28, 36–53, 55, 61, 62, 64–68, 70, 73, 74, 76–80, 82, 84, 85, 87, 88, 90, 95–97] [57, 59, 60, 92, 93]
Translation	4	(6)	[53, 56, 68, 84]
Derivation of themes	62	(91)	[19–21, 27, 28, 36–55, 61–71, 73–75, 77–80, 82–85, 87, 88, 90, 95–98] [57–60, 91–94]
Use of software	41	(60)	[19–21, 28, 36, 38, 40–48, 52, 54, 55, 61, 62, 64, 65, 67, 71, 72, 74, 77, 78, 80, 82–84, 87, 89, 96, 97] [57, 59, 60, 92, 93]
Participant feedback or member checking	10	(15)	[37, 54, 63, 78, 84, 85, 88] [57, 60, 91]
**Reporting:**			
Participant quotations provided	66	(97)	[19–21, 27, 28, 36–56, 61–90, 96–98] [57–60, 91–94]
Range and depth of insight into prostate cancer screening	59	(87)	[19–21, 27, 28, 36, 38–56, 61–68, 70, 71, 73–79, 81–86, 88–90, 96–98] [58, 60, 91, 92, 94]

### Synthesis

**Fig 2:**
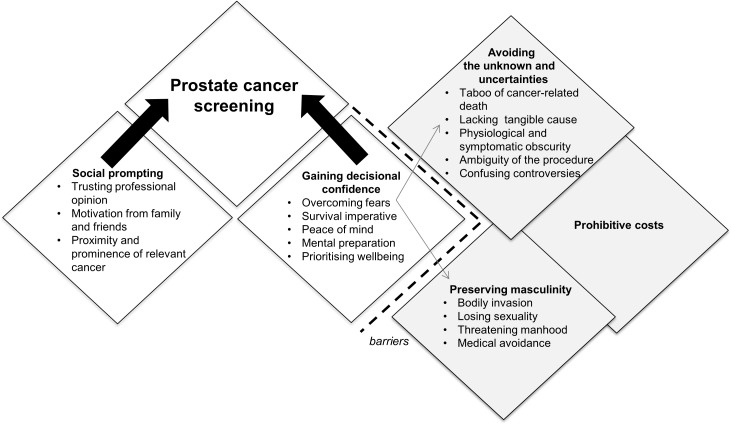
Thematic schema.

We identified five themes on men’s perspectives on prostate cancer screening: social prompting, gaining decisional confidence, preserving masculinity, avoiding the unknown and uncertainties, and prohibitive costs. The respective subthemes are described below. Table 3 provides selected participant quotations from the included studies, and indicates the studies that contributed to each theme. A thematic schema to illustrate the conceptual links among themes is provided in Fig 2.

**Table 3:** Illustrative quotations.

Theme	Source text	Contributing studies
**Social prompting**		
Trusting professional opinion	So I tend to leave the expert to do their job. I explain what’s wrong with me, and I hope and trust that if they’re well qualified, that I’m going to get the right treatment.[83] My doctor talked to me about prostate cancer and the test. He said, “You’re a Black male, you’re getting closer to the age, and that you need to have the test done.” So I did it…I’m around a lot of medical people around this area, so it’s the environment that’s comfortable to me right now.[47] It’s nice to have the information but sometimes. . . it’s too much information. . . it can put you on the worry, I feel quite happy that uh I had the blood test and if there was anything wrong I have always put my faith in doctors and the Health Service.[52]	[19, 27, 28, 38, 47, 49, 52, 54, 78, 81, 83] [59, 60, 91, 93]
Motivation from family and friends	I had a friend of mine who stayed on me about taking the prostate cancer test. Yeah, constantly, every time I would see her she used to ask me if I had got the test done yet. I would say, “No,” but I finally got it done because of her. I felt kind of embarrassed at first. You know how men are. But now I don’t feel that way anymore. I used to feel embarrassed, but now it’s just a test… I really thank her for sticking by me. My wife, she was with me too, though.[47] I told him, ‘Either I make the appointment for you again or you make it,’ and then, of course, ‘Give me the doctor’s number because I’m going to check to see if you made it.’[55]	[40, 41, 47, 52, 55, 56, 65, 69, 71, 72, 74, 77, 79, 83, 99] [91] [57, 92]
Proximity and prominence of relevant cancer	I do it because I lost my mother to cancer; I lost my father to cancer so just for me personally, you know, if I can find out something before it’s out of control I want to know about it.[42] I think how that might have impacted me personally, might influence how you would accept that because I have an uncle who was diagnosed with testicular cancer. . . . In 2000, my baby sister died from breast cancer. I have a sister-in-law who’s a seven-year survivor. . . . It has impacted me personally [and that] carries more weight.[66] After I see what my father went through… I will do anything that is necessary to keep me in health.[51]	[21, 42, 47, 51, 64–66, 69, 83, 85, 100] [60, 91]
**Gaining decisional confidence**		
Overcoming fears	You got to go to have this [DRE]. So it became a psychological game that I played with myself. You know, go up in there and get that done because if you don’t, it may be the end.[19] I’ve had the exam which is embarrassing and everything, but, I usually grit my teeth and go on and go through it because of the fact that it (prostate cancer) can be serious.[81] But it was just the thought that I'm going to have to do this. You know what I say-I encourage that if you have not had it you just grit your teeth and go on and get it done.[27]	[19, 27, 55, 61, 74, 81, 82]
Survival imperative	It were nearly three weeks waiting. . .your mind’s thinking all things then. . . the big C, but if you’ve got it you want to start treatment as early as possible, and if you haven’t you want to know as early as possible so you can settle your mind.[36] I honestly believe the knowing, and having the option of prevention, outweighs all the other risks. . . risk doesn’t matter, you gotta do the proper things for health anyway.[77] That [screening] is the one way to know to prevent it [prostate cancer], because they say that if you can discover it earlier you can cure it, but if it’s too late then all you can say is goodbye.[85]	[20, 21, 28, 36, 42, 43, 54, 71, 74, 77, 79, 85] [60, 91, 92]
Peace of mind	It puts your mind at ease.[39] I think my odds of developing it are probably better than most, but I think the fact that I’m coming here to get checked makes me feel a lot better.[48] It was a win-win situation. . . if I had no signs of prostate cancer that’s great, I’m reassured. If I have, I’ll get early treatment. . . so I can’t go wrong.	[36, 39, 48, 52, 54, 71, 79, 92]
Mental preparation	I would usually worry in between the PSA test and getting the results that is when I would work myself up into a right state. [79] You should prepare yourself psychologically to get the test done.[79]	[49, 79]
Prioritising wellbeing	I’ve been blessed with good health, for the most part, and I just did not want to run the risk. I didn’t want to do something stupid. . . if there’s a test, or an exam or something, I’m going to take it. . . I just want to be preventive, instead of regretting after the fact. [77] I have no problem with you know cause I’m–I’m curious, I want it done, I want to know what—what would be my life span you know and that going to hinder me I need to go ahead and get it done.[54] I was listening to something, and they were saying that all black males over 50 need to be tested. I’m not saying wait until you get 50 because you might not make it to 50. You’re right. You might not make it there. I’m saying if you feel funny in your 20s you had better go… it won’t hurt nothing. Cause I don’t see no harm in it.[69]	[19, 21, 27, 36, 38, 47, 54, 55, 65, 69, 74, 77, 80, 81]
**Preserving masculinity**		
Bodily invasion	I think that it is the fact of going through that physical test and it feel like it invades my privacy.[27] There should be other ways to determine or detect other than an invasive procedure like that because I tell you the rectal exam is invasive, I felt degraded.[81] I think most men are reluctant to take the exam (rectal) it’s like an invasion of your privacy, you have to overcome this issue of someone probing up your rectum, it's embarrassing. . .I don't feel that the embarrassment is the reason not to be tested. I’m eating and exercising, but you know one really doesn’t like a rectal exam, but if I need it I’ll get it done.[82]	[20, 27, 36, 41, 42, 53, 55, 61, 62, 69, 78, 81, 82, 84, 87, 88, 97, 100] [60, 94]
Losing sexuality	One of the things they may think of is the inability to perform sexually, and for a male that's devastating and for a female too.[27] Most Black men fear that if they have it done, it might affect their sexual performance. [78] Some blokes think if they have a prostate problem, your prostate gets removed and that’s the end of your sex life. So there are a certain group of people who think, bugger this, I’m still performing, I’ll take it right to the wire.[72]	[19, 20, 27, 41, 65, 68, 71–74, 77, 78, 84, 100] [94] [57, 58, 60, 92]
Threatening manhood	Most men probably tend to shy away from it because of those kinds of things (feeling of being violated). Most men have this macho ideal man I’m not going to let the doctor do that to me.[69] We have to be [fearful] when it comes to our manhood. For the Blackman, anything that got something to do with their manhood, they shy away from if it.[42] You hear stuff about men [who] are more private about their health and they do not talk about it much. Maybe with men, being unhealthy is a sign of being unmanly, so maybe we are not as likely to talk about it with each other.[66]	[19, 40–42, 51, 53, 55, 61, 64–66, 69, 70, 72, 74, 76, 78, 81, 82, 84, 88, 89, 96, 97, 99–101] [94] [57, 60]
Medical avoidance	Many black men do not go for regular check-ups. They do not check on themselves. They may be in pain, but they are like, oh it will go away. We as blacks, period. . . we let things get worse before we go see about it.[27] Nobody is worried and nobody goes for regular checks. It would be a prevention to go and see the doctor just for a check-up, like women do for breast cancer to make sure that everything is fine.[37] You don’t go to the doctor unless you were sick or you felt bad. It wasn’t a common practice to just go to the doctor for a check-up or physical unless you were going on a job or you had to have one or something like that. It was just a fact.[88]	[19–21, 27, 28, 37, 42, 45, 53, 55, 64, 69, 70, 72, 79, 86, 88, 89, 92, 97, 101]
**Avoiding the unknown and uncertainties**		
Taboo of cancer-related death	After four heart attacks, a couple of angioplasties, a couple of stents, and a four-way bypass, I’ve had so many things go wrong with me, I don’t want to find out if there’s anything else wrong. I really don’t want to know.[62] Well there’s also fear of finding out that you’ve got cancer … Fear of the unknown. That’s why a lot of people ain’t going to get themselves checked out.[42] People are scared about it [cancer], and they don’t want to know. So it’s almost like if they don’t know about it, it doesn’t exist.[41]	[19, 20, 28, 36, 37, 39, 41, 42, 54–56, 62, 77–79, 88, 92, 96, 99–101]
Lacking tangible cause	I just feel real healthy. I work out a lot, take a lot of supplements, and I just don’t feel the risk of that. And I also read years ago that if you’re very sexual, it limits the amount of back-up in your prostate area, so everything’s been working for me well. If there’s no history of it in your family, and my brother got checked out and he’s fine, and there’s no signs, and I eat right, and I don’t see a close friend or relative get it, I’m just going to put it off.[41] It's a blokey thing to think to yourself, well, I am healthy, if it's not affecting me, why bother with it? If it's not broke, don't try to fix it—don't touch it. Blokes tend to put it aside, try to ignore it, or pretend it's not there until such time as they absolutely have to do something about it.[86]	[20, 36, 43, 50, 58, 62, 64, 69, 74, 76, 82, 84–86, 89, 99]
Physiological and symptomatic obscurity	The last few years we’ve been hearing about prostate cancer more and more like and all we think is oh I don’t want that. But we don’t know what the gland’s there for; we don’t know what it does, so we don’t know what to do.[44] I didn’t know much about it. As I said I didn’t even know women didn’t even get it, so that’s pretty ignorant.[46] But that cancer is for old men and we are still young, so why should we waste time and money going for check-ups.[50]	[39, 44–46, 50, 54, 55, 88, 89, 100]
Ambiguity of procedure	My fear was mostly that it would be painful.[88] I thought it [PSA] was an invasive test. And I think that’s off-putting.[36]	[36, 41, 42, 53, 54, 65, 70, 88, 91]
Confusing controversies	I’ve had the PSA test done a couple of times. My understanding of that is that’s not necessarily a reliable indicator of whether you’ve got it or not. So there are doubts surrounding the test itself. . . which is not that reassuring.[46] He did explain that although the test would find out whether I had prostate cancer, it wasn’t the be all and end all as far as the test was concerned. In other words if I came up as clear, there still may be some signs there, which I thought was a little strange at the time.[83]	[21, 36, 38, 46, 52, 62, 65, 69, 83, 92, 93, 95]
**Barriers to access**		
Prohibitive costs	I think another reason (why men don’t get tested for prostate cancer) is. . . health insurance. I think that more black males don’t have access to health insurance than white men. We cannot afford getting the test.[27] We have too many survival issues in our community. I am not worried about my health, I'm worried about money.[89] A lot of people don’t have health insurance, they don’t have adequate health insurance. So, what people do a lot of times is just don’t deal with those situations that cause them to have to spend money unless it’s an absolute emergency ‘cause we don’t have the health insurance and I don’t know whether it’s a myth or reality, but you can’t walk into a health center and get a prostate exam for free.[80]	[19, 20, 27, 40, 54, 60, 64, 80, 89, 90]

#### Social prompting

Trusting professional opinion–Some participants had “confidence in doctors” to initiate and make recommendations about prostate cancer screening.[19, 27, 28, 38, 47, 49, 52, 54, 59, 60, 78, 81, 83, 91, 93] Many felt overwhelmed by the complicated medical information and thus did not feel competent enough to “evaluate the pros and cons or the risks” on their own.[38, 52] Having a strong relationship with their health care provider allowed participants to make this important health decision for them.[78]

Motivation from family and friends*–*Family and friends motivated men to be screened for prostate cancer.[40, 41, 47, 55–57, 71, 72, 79, 83, 91, 92, 99] Participants appreciated their spouses’ encouragement, their constant “nagging”[79] to pursue screening and “for sticking by”,[47] during the decision making and screening process. Having a family member who had prostate-related problems (e.g. benign prostatic hyperplasia) also prompted them to undergo screening, as they “wouldn’t have started checking”[69] otherwise.[65, 69, 83, 91, 98]

Proximity and prominence of cancer*–*Participants who had seen the devastating impact of cancer among their family or friends felt acutely aware of cancer, and were prompted to undergo prostate cancer screening.[21, 42, 47, 51, 60, 65, 66, 69, 75, 83, 85, 91, 100] The death of family members due to cancer and “seeing what [they] went through”[51] as well as understanding the potential of cancer “running in the family,”[66] caused participants to become proactive about cancer screening, including for prostate cancer. Some believed their family members had cancer detected “too late”.[75]

#### Gaining decisional confidence

Overcoming fears*–*Preparing to screen for prostate cancer was described by participants as a “psychological game”[19] that required them to overcome the stigma surrounding the screening procedure.[19, 27, 55, 61, 81, 82] Prostate cancer screening was initially perceived as “embarrassing,”[81] “uncomfortable,”[82] and “dreaded,”[61] which was later on accepted as “necessary,” [82] and “routine.”[61]

Survival imperative–Improving their chance of survival through early detection motivated men to undergo prostate cancer screening.[20, 21, 28, 36, 42, 43, 60, 71, 74, 77, 85, 91, 92] Early detection was perceived as imperative to prevent severe morbidity or mortality, so they could continue to live a “normal life.”[21] They believed that cancers detected early had a “higher chance”[20] of cure.

Peace of mind*–*The possibility of prostate cancer was often “constantly nagging at the back of their mind”[79] particularly for men with increased risk due to family history of the cancer. Some participants believed they had “nothing to lose”[36] by going ahead with the screening. By doing so, they felt reassured and their “mind was at ease.”[36, 39, 48, 52, 71, 79, 92] Prostate cancer screening provided “peace of mind for family and loved ones too.”[71]

Mental preparation*–*Preparing for the procedure of screening was psychologically confronting and overwhelming for men.[19, 49, 79] The fear of having an invasive procedure required men to be mentally prepared as they were hesitant to commit.[79] [19, 79] After the test, many men felt “anxious”[49] awaiting the results and left with a “great void of uncertainty”[79] as they were terrified to receive a positive result.

Prioritising wellbeing*–*Men valued survival and family: “I want to live as long as I can and be around for my children and grandchildren.”[47] For those who had not experienced severe health problems, they “did not want to run the risk”[77] and instead “wanted to be preventive, instead of regretting”[77] their decision in the future. Some regarded prostate cancer screening as a “win-win situation”[36], and regardless of the outcome, they could “not go wrong.”[36] Some men strived to maintain a sense of control of their body, and were “curious”[54] and wanted to know “what is really going on in my body”[54], and thus were inclined to participate in prostate cancer screening.

#### Preserving masculinity

Bodily invasion*–*Some men felt a loss of personal dignity after undergoing the invasive procedure of screening.[20, 27, 53, 55, 60, 61, 81, 82, 88, 94, 97, 100] For men who had not previously been screened for prostate cancer they were fearful of the procedure and did not like the idea of a doctor “messing with me there.”[87] For men who had participated they felt the procedure was “violating”[97] and some “felt invaded,”[27, 81, 82, 97] and therefore were unwilling to complete the screening again.[78, 82] Some persevered through the unpleasantness of the procedure by focussing on the necessity–“if I need it I’ll get it done”.[82]

Losing sexuality*–*Men were fearful that consequences of screening may cause sexual dysfunction,[19, 20, 27, 57, 58, 60, 65, 68, 71–75, 77, 78, 92, 94, 100] and this was a “major concern.”[19] Being unable to perform sexually would be “devastating.”[27] Prostate cancer and subsequent prostatectomy was seen as “taking the manhood away”[71] and thus men were reluctant to undergo screening. They wanted to avoid diagnosis of prostate cancer as they did “not want to end up impotent,”[77] which some had observed in other men who had been treated for prostate cancer.

Threatening manhood*–*Being diagnosed with prostate cancer was perceived to potentially jeopardise a man’s manhood or masculinity.[19, 39, 41, 42, 51, 53, 55, 57, 60, 63–66, 69, 70, 72, 74, 75, 78, 82, 84, 88, 89, 94, 96, 97] Their “macho man”[53] image would be compromised, as “being unhealthy is a sign of being unmanly.”[66] Some men were uneasy and reluctant to be screened as the DRE was perceived to have “homosexual implications.”[19, 51, 69, 74, 89] Some also feared being diagnosed with prostate cancer as they would have to relinquish their role as “the rock”[66] of their family.

Medical avoidance*–*Some participants were generally averse to interacting with the medical system, particularly if it was seen as unnecessary given the absence of symptoms or perceptible health problems.[19–21, 27, 28, 37, 42, 45, 53, 55, 63, 64, 69, 72, 86, 88, 89, 92] They reasoned that “if it don’t hurt don’t fix it.”[20] Even after been advised by a doctor to undergo further tests or examinations they would “postpone and postpone”[28] as they refused to be seen as vulnerable and were terrified of potentially receiving bad news. Some men believed that it was “not socially acceptable”[69] to go to the doctor like women,[55, 64, 69, 70, 86] which they attributed to their macho mentality[21, 63, 72]–that they were “tough”[72] and “infallible.”[21]

#### Avoiding the unknown and uncertainties

Taboo of cancer-related death*–*Fears of being potentially diagnosed with prostate cancer and dying caused men to ignore the matter.[19, 20, 28, 36, 37, 39, 41, 42, 54, 56, 62, 63, 77–79, 88, 92, 96, 99, 100] In one study, African American men felt that “anything that a Black man thinks is gonna kill him, he ain’t gonna want to talk about”.[41] Cancer was considered to be “a death sentence,”[28] and men expressed that they would “rather not know”.[27] For men who had previous or ongoing health problems, they were particularly concerned that another diagnosis i.e. of prostate cancer would increase the burden of living with the disease to beyond what they could cope with.

Lacking tangible cause*–*The absence of signs and symptoms made it difficult for men to understand the value of screening.[20, 36, 43, 50, 58, 62, 64, 69, 74, 84–86, 89, 98] Some felt they were healthy and were convinced that “if it’s not affecting me, why bother with it?”[86] as there was “nothing wrong…no pain or disease.”[50]

Physiological and symptomatic obscurity–Although participants indicated their awareness of prostate cancer, some did not know “what the gland’s there for, we don’t know what it does, so we don’t know what to do.”[44] One participant highlighted that he was “pretty ignorant“[46] as he “didn’t even know women didn’t get it.”[46] Some younger men felt there was no need to spend time and money for screening as prostate cancer was an “old man”[50] cancer. Further, participants had lack of awareness of the symptoms of prostate cancer.

Ambiguity of the procedure*–*Some men described having a vague understanding of the procedures of prostate cancer screening, which caused them to feel concerned and anxious about undergoing screening.[36, 42, 54, 65, 88, 91] Screening was at times acted upon because they received instruction by their doctor, although they had little knowledge regarding the PSA testing.[91] Due to lack of knowledge and awareness, some men stated there was “too much confusion” and assumed that all prostate cancer screening was “invasive”[36] and “painful.”[88] However, some became aware that a blood test was available, and were relieved that they would not have to undergo a DRE.[36]

Confusing controversies*–*The controversies regarding the accuracy of prostate cancer screening caused some men to feel confused and uncertain.[21, 36, 38, 46, 52, 62, 65, 67, 69, 83, 92, 95] They questioned “what’s the point of getting a test if it might not be reliable?”[62] For others, they found the procedure “not that reassuring,”[46] as there was still a chance they could have prostate cancer. Thus, decision making was complex as they had to “evaluate the pros and cons”[38] in the context of such uncertainties. Some men also disclosed that the potential risks involved in prostate cancer screening were not addressed by doctors, despite highlighting the benefits of the procedure. [93]

#### Prohibitive costs

The cost of prostate cancer screening created difficulty for men seeking participation[19, 20, 27, 40, 54, 60, 64, 80, 89, 90] as many were not “able to afford the doctors.”[40] However, concerns regarding costs were only identified among African American and African-Caribbean participants in the United States. In 14 studies involving African American men, men disclosed that they could not afford health insurance and therefore were unable to be screened for prostate cancer despite their increased risk.[19, 20, 27, 39, 40, 54, 62, 64, 71, 73, 76, 80, 87, 89] African-Caribbean men also articulated that “health insurance is probably the biggest impediment”[60] for men to regularly partake in screening.

## Discussion

Our findings demonstrate that men’s expectations and values regarding prostate cancer screening reflects a complex decisional matrix. Active recommendation to screen from health care providers, persuasion from their close social networks, and exposure to personal experience of cancer supported men’s acceptance and willingness to undergo prostate cancer screening. Further, men believed that early detection could improve chances of survival, which was also a strong motivator to participate in screening. However, some men refused or delayed prostate cancer screening as risks such as erectile dysfunction were regarded as a threat to their masculinity and bodily dignity, Also, men were reluctant to contemplate mortality as it was too confronting (or perceived as irrelevant by younger men), or were anxious about the unknowns and uncertainties of the procedure and the potential distress if screened positive for prostate cancer.

Our review also highlights differences in men’s perspectives of prostate cancer screening by age, ethnicity and family history of prostate cancer. Some younger men felt they had less knowledge about the risk of prostate cancer, and assumed that screening was not relevant at their age as prostate cancer was thought to be more prevalent in older men. Although African American men have an increased risk of prostate cancer, they felt their knowledge of the disease was limited compared to other populations, and identified cost as a major barrier to screening. Many men who had a family history of prostate cancer were acutely aware of the consequences on health and quality life, and were thus motivated to participate in screening. However, we did not identify any clear differences by country.

The decision making process for prostate cancer screening align with the domains of the Integrated Behaviour Model, which has been commonly used to explain decision making in cancer screening.[102, 103] The model theorises that intentions (founded by attitudes, subjective norms and perceived self-efficacy) are the predominant determinants of enacting behaviour; while ability, environmental constraints and habit directly enable or constrain the resulting action.[104, 105] Thus, our findings demonstrate that men’s decision making for prostate cancer screening is influenced by knowledge obtained through support networks and other means, the personal impact of knowing someone who has suffered from cancer, and their motivation to maintain their wellbeing. However, the only environmental constraint identified in the review was the out-of-pocket costs of screening, which varied by jurisdiction.

Our findings are consistent with similar studies for other cancers. Receiving a direct recommendation from a health care provider to undergo screening has also been found to prompt and reinforce motivation for participation in bowel,[106, 107] breast,[108–110] and cervical cancer screening.[111, 112] Other facilitators of screening also include early detection and prevention of cancer,[109, 112, 113] peace of mind,[114–116] and being in control of own health.[113, 114] Studies have also shown that patients are conscious of their own lack of awareness about the risk and harms of screening, which contributes to uncertainties in decision making.[117–119] In terms of barriers, concerns about invasion of privacy in screening have also been documented in the context of cervical cancer.[120] In prostate cancer screening, sexual impotence was identified as a major concern in our study. However in contrast, findings from a discrete choice experiment suggests that the risk of erectile dysfunction did not significantly influence men’s preferences for screening, and men were more willing to accept this potential harm to avoid prostate cancer related death.[121]

Our review involved a systematic and comprehensive literature search, and we synthesized 68 qualitative studies, with 3,029 participants from nine countries to develop a new analytical framework reflecting the complexities in decision-making about prostate cancer screening among men. We also conducted an independent assessment of the transparency of study reporting. However, there are some potential limitations. Although one author coded the studies, the preliminary findings and coding were discussed with multiple investigators who had read the papers, to enhance the analytical framework and to ensure that the full spectrum of perspectives reported in the primary studies were included in the final analysis. We excluded non-English articles to minimise cultural and linguistic misinterpretation. Also, we were unable to delineate differences between types of screening modality as these were not specified in most studies, and whether perspectives varied according to whether men had actually experienced screening.

The lack of understanding about the process and risk of screening, and the potential threat to masculinity as a consequence of further interventions if screened positive, compound the complexities of men’s decision-making about prostate cancer screening. This suggests the need for accessible and comprehensive information about the screening procedure, the potential benefits and harms of screening; particularly as shared decision making about prostate cancer screening is still not being widely implemented in primary healthcare settings.[122] Current guidelines for prostate cancer screening recommend against prostate cancer screening or suggest a shared decision making approach that address the benefits and harms for specific ages or high risk groups.[11, 14–16] They also suggest consideration of men’s preferences and values; however they do not explicitly mention the values that are important to men which were disclosed in our review, and includes invasion of privacy, masculinity and impotence.

Decision aids may be beneficial for presenting population based evidence on benefits and harms of screening, but implementation can be challenging. Randomised controlled trials have shown that decision aids can improve informed decisions, communication between patients and health can providers, and therefore facilitate informed decision making.[123–125] Further, trials specific to prostate cancer screening, have found decision aids improve knowledge of PSA testing and reduced men’s desire to undergo screening.[126] However, at present, information on the harms of overdiagnosis and overtreatment is rarely presented.[127] Further, decision aids have not been widely implemented,[128] due to barriers including insufficient practitioner time, unawareness of and access to decision aids, and possibly lack of skills to utilise with patients in a clinical environment.[129–132]

Our findings demonstrate that participation in prostate cancer screening is informed by an individual’s subjective norms. Although routine screening for prostate cancer screening is not recommended, many men participate and remain enthusiastic about screening, as they believe that early detection prevents cancer.[133] In light of the potential harms of screening, addressing individual and community perceptions of prostate cancer screening through communication and health messages is important to normalise the decision to forgo prostate cancer screening.[134]

Therefore we suggest further work to gain understanding about what men understand about overdiagnosis as this does not appear to be explicitly addressed in existing studies. This could include eliciting their attitudes, reactions and choices made when provided with such information, and the harms and risks men prioritise, and how these potentially impact the decision making process, as has been done in the context of breast cancer.[135] As in other previous work in breast cancer screening,[134] assessing the impact of recent policy changes to prostate cancer screening uptake and understanding awareness of the guidelines changes, the controversy, and what formulates decision making is imperative to develop interventions that can improve the decision making process regarding prostate cancer screening.

As clinician recommendation is an important contributor to prostate cancer screening decisions, an understanding of how pre-screening discussions are conducted in clinical practice is important. Future studies are needed to promote shared decision making about prostate cancer screening. Further, research regarding shared decision making should focus not only on shared decision making as an outcome but also through understanding the processes of formulating a decision, delivering of information of the procedure and associated benefits and harms, and patient’s emotional experience and attitudes towards the decision making process. This will facilitate identification of best practices to disseminate shared decision making interventions.

Prostate cancer screening is sourced and accepted by men in order to prevent cancer-related morbidity and mortality and gain reassurance about their health. Their willingness to screen is informed by their primary care providers and support networks. However fears regarding their masculinity and invasiveness of the procedure, and hesitation relating to the ambiguities of the effectiveness, necessity and costs of screening, also contribute to their decision-making. Addressing the concerns and priorities of men regarding prostate cancer screening may empower men to make more informed decisions that reflect their personal priorities, preferences and values, and improve patient satisfaction and outcomes.

**Transparency declaration:** LJJ affirms that this manuscript is an honest, accurate, and transparent account of the study being reported; that no important aspects of the study have been omitted; and that any discrepancies from the study as planned (and, if relevant, registered) have been explained.

## Supporting information

S1 Text Search strategy. (DOCX)

S1 Table Study characteristics. (DOCX)

S2 Table ENTREQ checklist. (DOCX)

S3 Table PRISMA checklist. (DOC)
