# Supplementary material for: Men’s perspectives of prostate cancer screening: A systematic review of qualitative studies
Source: PLoS One. 2017 Nov 28;12(11):e0188258. doi: 10.1371/journal.pone.0188258 (PMC5705146; doi:10.1371/journal.pone.0188258)
Supplement: S1 Text — (DOCX) [file pone.0188258.s001.docx]

**S1 Text. Search strategy**

**Medline (since 1946)**

| 1 | communit$.tw. |  |
| --- | --- | --- |
| 2 | public$.tw. |  |
| 3 | men/ or exp population groups/ or women/ |  |
| 4 | Adult/ |  |
| 5 | or/1-4 |  |
| 6 | exp Prostate-Specific Antigen/ |  |
| 7 | prostate cancer screening.tw. |  |
| 8 | digital rectal$.tw. |  |
| 9 | (prostat$ and screen$).tw. |  |
| 10 | (prostat$ and detect$).tw. |  |
| 11 | (PSA screening$ or PSA test$).tw. |  |
| 12 | or/6-11 |  |
| 13 | 5 and 12 |  |
| 14 | exp Qualitative Research/ |  |
| 15 | qualitative$.tw. |  |
| 16 | exp Public Opinion/ |  |
| 17 | interview$.tw. |  |
| 18 | focus group$.tw. |  |
| 19 | (belief$ or attitud$ or perspective$).tw. |  |
| 20 | exp Attitude to Health/ |  |
| 21 | exp Health Knowledge, Attitudes, Practice/ |  |
| 22 | ethnograph$.tw. |  |
| 23 | phenomenol$.tw. |  |
| 24 | symbolic interact$.tw. |  |
| 25 | (thematic$ or theme$).tw. |  |
| 26 | grounded theory.tw. |  |
| 27 | or/14-26 |  |
| 28 | 13 and 27 |  |

**Embase (since 1980)**

| 1 | community$.tw. |  |
| --- | --- | --- |
| 2 | public$.tw. |  |
| 3 | adult/ |  |
| 4 | male/ |  |
| 5 | exp population/ |  |
| 6 | or/1-5 |  |
| 7 | exp Prostate-Specific Antigen/ |  |
| 8 | prostate cancer screening.tw. |  |
| 9 | digital rectal$.tw. |  |
| 10 | (prostat$ and screen$).tw. |  |
| 11 | (prostat$ and detect$).tw. |  |
| 12 | (PSA screening$ or PSA test$).tw. |  |
| 13 | or/7-12 |  |
| 14 | 6 and 13 |  |
| 15 | exp Qualitative Research/ |  |
| 16 | qualitative$.tw. |  |
| 17 | exp public opinion/ |  |
| 18 | exp attitude to health/ |  |
| 19 | (attitude$ or belief$).tw. |  |
| 20 | focus group$.tw. |  |
| 21 | interview$.tw. |  |
| 22 | ethnograph$.tw. |  |
| 23 | phenomenol$.tw. |  |
| 24 | symbolic interact$.tw. |  |
| 25 | (thematic$ or theme$).tw. |  |
| 26 | grounded theory.tw. |  |
| 27 | or/15-26 |  |
| 28 | 14 and 27 |  |

**PsycINFO (since 1806)**

| 1 | prostate cancer screening.tw. |  |
| --- | --- | --- |
| 2 | digital rectal$.tw. |  |
| 3 | (prostat$ and screen$).tw. |  |
| 4 | (prostat$ and detect$).tw. |  |
| 5 | (PSA screening$ or PSA test$).tw. |  |
| 6 | or/1-5 |  |
| 7 | community$.tw. |  |
| 8 | public$.tw. |  |
| 9 | (men$ or male$).tw. |  |
| 10 | population$.tw. |  |
| 11 | or/7-10 |  |
| 12 | 6 and 11 |  |
| 13 | exp Qualitative Research/ |  |
| 14 | qualitative$.tw. |  |
| 15 | (opinion$ or attitud$ or belief$ or perspectiv$).tw. |  |
| 16 | interview$.tw. |  |
| 17 | focus group$.tw. |  |
| 18 | ethnograph$.tw. |  |
| 19 | phenomenol$.tw. |  |
| 20 | symbolic interact$.tw. |  |
| 21 | (thematic$ or theme$).tw. |  |
| 22 | grounded theory.tw. |  |
| 23 | or/13-22 |  |
| 24 | 12 and 23 |  |

**CINAHL**

MH Prostate cancer screening

Limiters - Clinical Queries: Qualitative - Best Balance
