# Supplementary material for: Men’s perspectives of prostate cancer screening: A systematic review of qualitative studies
Source: PLoS One. 2017 Nov 28;12(11):e0188258. doi: 10.1371/journal.pone.0188258 (PMC5705146; doi:10.1371/journal.pone.0188258)
Supplement: S1 Table — (DOCX) [file pone.0188258.s002.docx]

**S1 Table. Characteristics of included studies**

| **Study** | **n*** | **Age range** | **Ethnicity** | **Population** | | **Methodology** | **Data Collection** | **Data Analysis** | **Topic** |
| --- | --- | --- | --- | --- | --- | --- | --- | --- | --- |
|  |  |  |  | **Screened** | **Not screened** |  |  |  |  |
| **Australia** | | | | | | | | | |
| Drummond 2001[^89^](#_ENREF_89) | 20 | NS | Italian Australians | NS | ⦁ | Qualitative | Semi structured face to face interviews & focus groups | Reflexive analysis | Knowledge, information sources |
| Ilic 2005[^63^](#_ENREF_63) | 33 | 45-89 | Unspecified | NS | NS | Grounded theory | Semi structured focus groups | Grounded theory, thematic analysis | Health attitudes, information seeking |
| Ilic 2013a[^90^](#_ENREF_90) | 12 | 35-56 | Unspecified | ⦁ |  | Qualitative | Interviews | Thematic analysis | Knowledge, awareness, perceptions, |
| Ilic 2013b[^95^](#_ENREF_95) | 76 | NS | Unspecified | ⦁ | ⦁ | Qualitative | Focus groups | Thematic analysis | Knowledge, information seeking and decision making |
| Madjar 2007[^91^](#_ENREF_91) | 71 | 18-80 | Unspecified | NS | NS | Qualitative | Interviews and focus groups | Thematic analysis | Individual and community perceptions |
| Pinnock 1998[^52^](#_ENREF_52) | 151 | NS | Unspecified | ⦁ | ⦁ | Qualitative | Focus groups | NS | Urological health issues of concern. |
| **Barbados** | | | | | | | | | |
| Ng 2013[^86^](#_ENREF_86) | 33 | 38-79 | African-Barbadian | NS | NS | Qualitative | Face to face interviews | Thematic analysis | Knowledge, access to health care and fears, beliefs, experiences. |
| **Belgium** |  |  |  |  |  |  |  |  |  |
| Engelen 2016 | 43 | 52-78 | Unspecified | ⦁ | NS | Qualitative | Semi structured focus groups | Thematic analysis | Views on decision aids for early detection |
| **Canada** | | | | | | | | | |
| Matthew 2011[^74^](#_ENREF_74) | 15 | 39-85 | Unspecified | ⦁  15 | ⦁ | Qualitative | Interviews | Phenomenological analysis | Factors that influence the formulation of risk perceptions |
| **Germany** | | | | | | | | | |
| Hannover 2010[^71^](#_ENREF_71) | 83 | 45-85 | Unspecified | ⦁ | ⦁ | Qualitative | Semi structured, face to face interviews | Content analysis | Barriers to screening. |
| **Nigeria** |  |  |  |  |  |  |  |  |  |
| Enaworu 2016 | 10 | 40-60 | Nigerian | ⦁ |  | Qualitative | Semi structured interviews | Thematic analysis | Factors that influence decision making |
| **Uganda** | | | | | | | | | |
| Nakandi 2013[^93^](#_ENREF_93) | 545 | 18-71 | Unspecified | ⦁  15 | ⦁  490 | Mixed-methods | Focus groups | Thematic analysis | Knowledge, attitudes, practices. |
| **United Kingdom** | | | | | | | | | |
| Avery 2008[^69^](#_ENREF_69) | 58 | 50-69 | Unspecified | ⦁ | ⦁ | Grounded theory and ethnography | Semi structured telephone and face to face interviews | Thematic Analysis | Decision making |
| Bancroft 2015[^48^](#_ENREF_48) | 26 | 40-69 | Unspecified | ⦁ |  | Mixed-methods | Open ended Interviews | Thematic analysis | Family history, psychological impact |
| Evans 2007[^38^](#_ENREF_38) | 28 | 40-75 | Unspecified | ⦁ | ⦁ | Qualitative | Semi structured interviews | Thematic analysis | Understanding, views, experiences |
| George 2004[^135^](#_ENREF_135) | 12 | 50-59 | Unspecified | NS | NS | Phenomenology | Semi structured Interviews | Phenomenological analysis | Experiences |
| Horwood 2014[^94^](#_ENREF_94) | 21 | 52-72 | Unspecified |  | ⦁ | Qualitative | Interviews | Thematic analysis | Knowledge and attitudes, high risk men, prevention |
| Rai 2007[^42^](#_ENREF_42) | 20 | 45-75 | Unspecified |  | ⦁ | Qualitative | Semi structured, face to face interviews | Thematic analysis | Decision making, access to information |
| Seymour-Smith 2016 | 10 | 30-79 | African-Caribbean | NS | NS | Qualitative | Semi structured interviews | Synthetic discursive | Knowledge, barriers of DRE |
| **United States** | | | | | | | | | |
| Allen 2007[^19^](#_ENREF_19) | 51 | 35-70 | African American | ⦁ | ⦁ | Qualitative | Semi structured interviews and focus groups | Thematic content analysis | Perspectives and decision making |
| Blocker 2006[^27^](#_ENREF_27) | 29 | 34-68 | African American | ⦁ | ⦁ | Qualitative | Focus groups | Thematic analysis | Knowledge, beliefs, behaviour change and barriers |
| Bryan 2008[^98^](#_ENREF_98) | 28 | 35-69 | African/Caucasian-American |  | ⦁ | Qualitative | Focus groups | Content analysis | Increasing information seeking and recruitment for a program. |
| Clarke-Tasker 2002[^36^](#_ENREF_36) | 12 | 38-80 | African American | ⦁ | ⦁ | Qualitative | Focus groups | Content analysis | Knowledge, attitudes. |
| Conde 2011[^28^](#_ENREF_28) | 20 | 40+ | Filipino | ⦁ | ⦁ | Qualitative | Focus groups | Thematic analysis | Barriers and facilitators, identifying strategies. |
| Dale 1998[^67^](#_ENREF_67) | NS | 39-95 | African American | ⦁ |  | Qualitative | Focus groups | Content analysis | Health prevention behaviours, attitudes and practices |
| Dube 2005[^37^](#_ENREF_37) | 33 | 40-75 | Unspecified | ⦁ | ⦁ | Qualitative | Focus groups | Thematic analysis | Experiences and knowledge |
| Edwards 2000[^68^](#_ENREF_68) | 10 | 40-60 | African American | ⦁ | ⦁ | Mixed Methods | Semi structured, face to face interviews | Thematic analysis | Health seeking behaviours in Military |
| Farrell 2002[^70^](#_ENREF_70) | 40 | 40-65 | Caucasian/African American | ⦁ | ⦁ | Mixed Methods | Interviews | Grounded theory and content analysis | Decision making after counselling |
| Ferrante 2011[^92^](#_ENREF_92) | 64 | 50-69 | White, Hispanic, Black, Asian | ⦁ | ⦁ | Grounded theory | Interviews | Grounded theory | Decision making |
| Ford 2006[^73^](#_ENREF_73) | 21 | 55-87 | African American | NS | NS | Qualitative | Focus groups | Content analysis | Perceptions |
| Forrester-Anderson 2005[^20^](#_ENREF_20) | 104 | 40-80 | African American | ⦁ | ⦁ | Qualitative | Focus groups | Grounded theory | Knowledge, perceptions, attitudes and behaviours |
| Friedman 2009[^49^](#_ENREF_49) | 25 | 47-64 | African American | NS | NS | Qualitative | Interviews and focus groups | Thematic analysis | framework understanding and knowledge |
| Friedman 2012[^50^](#_ENREF_50) | 81 | 21-77 | African American | ⦁ | ⦁ | Qualitative | Focus groups | Thematic analysis | Practices, barriers and strategies to improve communication |
| Fyffe 2008[^61^](#_ENREF_61) | 24 | 22-85 | African American | ⦁ | ⦁ | Qualitative | Focus groups | Thematic analysis | Awareness, perceptions, problems and solutions |
| Gittens 2016 | 44 | NS | West Indian American, African American | NS | NS | Qualitative | Focus groups | Grounded theory | Cultural and familial influences |
| Griffith 2007[^84^](#_ENREF_84) | 66 | 35-83 | African American | ⦁ | ⦁ | Qualitative | Focus groups | Thematic analysis | Physical, cultural, social factors, decision making |
| Gwede 2015[^59^](#_ENREF_59) | 20 | 40-70 | Unspecified | ⦁ | ⦁ | Qualitative | Interviews | Content analysis and constant comparison method | Controversy knowledge, perceptions of informed decision making |
| Harvey 2011[^62^](#_ENREF_62) | 15 | 40-78 | African American | ⦁ | ⦁ | Qualitative | Focus groups | Thematic analysis | Contextual factors in attitudes and beliefs |
| Hicks 2014[^85^](#_ENREF_85) | 17 | 25-43 | Latino | ⦁ | ⦁ | Qualitative | Semi structured, face to face interviews | Grounded theory | Sons of men with prostate cancer, effects of familial communication |
| Hill 2013[^39^](#_ENREF_39) | 14 | 45-70 | African American | ⦁ | ⦁ | Qualitative | Semi structured, face to face interviews | Thematic analysis | Experiences, attitudes, behaviours, barriers, decision making |
| Hunter 2015 | 38 | NS | African American | NS | NS | Qualitative | Focus groups | Grounded theory | Perceptions, beliefs of risk and testing |
| Jones 2010[^40^](#_ENREF_40) | 17 | 41-71 | African American | NS | ⦁ | Phenomenology | Interviews | Iterative comparative analysis | Decision making process |
| Marks 2004[^79^](#_ENREF_79) | 49 | NS | African American | NS | NS | Qualitative | Focus groups | Thematic analysis | Knowledge, information needs, distrust |
| McFall 2003[^96^](#_ENREF_96)** | 90 | 50-57 | Hispanic, non-Hispanic white, African American | ⦁ | ⦁ | Qualitative | Focus groups | Thematic analysis | Reactions to outcomes, developing a decision aid, |
| McFall 2006[^21^](#_ENREF_21)** | 90 | 40-70 | African American, Hispanic, non-Hispanic white | ⦁ | ⦁ | Qualitative | Focus groups | Thematic analysis | Beliefs, risk, knowledge |
| McGovern 2004[^41^](#_ENREF_41) | 16 | NS | Unspecified |  | ⦁ | Qualitative | Focus groups | Thematic analysis | Experiences of false positive result |
| Meade 2003[^80^](#_ENREF_80) | 71 | 18-85 | Hispanic, African American | ⦁ |  | Qualitative | Focus groups | Thematic analysis | Screening knowledge, attitudes and beliefs, learning preferences. |
| Nash 2002[^58^](#_ENREF_58) | 37 | 39-67 | Black and white, urban and rural | ⦁ | ⦁ | Qualitative | Focus groups | Thematic analysis | Health concerns, health prevention, knowledge, attitudes, beliefs. |
| Ocho 2013[^64^](#_ENREF_64) | 75 | 19-60 | Trinidad and Tobago groups | ⦁ | ⦁ | Qualitative | Semi structured focus groups | Thematic analysis | Awareness, barriers, facilitators to accessing services. |
| Odedina 2004[^51^](#_ENREF_51) | 49 | NS | African-American | ⦁ | ⦁ | Ethnography | Focus groups | Thematic analysis | Factors and programs influencing screening behaviours |
| Oliver 2007[^75^](#_ENREF_75) | 9 | 43-72 | African-American | ⦁ | ⦁ | Qualitative | Semi structured, face to face interviews | Content analysis | Attitudes, beliefs |
| Owens 2016 | 39 | 37-66 | African American | NS | NS | Qualitative | Focus groups | Grounded theory | Risk and screening knowledge, decision making process |
| Richardson 2004[^81^](#_ENREF_81) | 15 | 33-47 | African American | ⦁ |  | Qualitative | Focus groups | Thematic analysis | Understanding of knowledge and barriers |
| Rivera-Ramos 2011[^76^](#_ENREF_76) | 10 | 48-66 | Latino | ⦁ | ⦁ | Qualitative | Semi structured, face to face interviews | Grounded theory | Culture, gender’s influence on attitudes, beliefs and behaviours. |
| Robinson 1996[^87^](#_ENREF_87) | 56 | NS | African American | ⦁ | ⦁ | Qualitative | Focus groups | NS | Attitudes, willingness to participate |
| Ross 2007[^99^](#_ENREF_99) | 52 | 35-79 | African American | NS | NS | Qualitative | Semi structured focus groups | Theory of reasoned action | Predictors information seeking behaviour |
| Sanchez 2007[^72^](#_ENREF_72) | 31 | 40-70 | African American | ⦁ | ⦁ | Qualitative | Semi structured focus groups | Descriptive analysis | Factors influencing decision making |
| Sutkowi-Hemsworth | 13 | 50-69 | Unspecified | NS | NS | Qualitative | Semi structured, face to face interviews | Thematic analysis | Perceptions of harms and benefits, decision making factors |
| Taitt 2015 | 13 | 40-75 | African-Caribbean | 12 | 1 | Phenomenology | Open ended interviews | Content analysis | Beliefs, perceptions of susceptibility, severity of disease |
| Taylor 2001[^97^](#_ENREF_97) | 44 | 40-70 | African American | NS | NS | Qualitative | Focus groups | Thematic analysis | Evaluation of education materials |
| Taylor 2013[^43^](#_ENREF_43) | 7 | 42-57 | African American | ⦁ | ⦁ | Mixed methods | Face to face interviews | Thematic analysis | Impact of community health centre on behaviour |
| Webb 2006[^53^](#_ENREF_53) | 32 | NS | African American | ⦁ | ⦁ | Qualitative | focus groups | Thematic analysis | Gender comparisons, attitudes, knowledge, beliefs |
| Winterich 2009a[^88^](#_ENREF_88) | 65 | 40-64 | African American and white | ⦁ | ⦁ | Qualitative | Semi structured, face to face interviews | Thematic analysis | Race, education, screening status impact on knowledge and beliefs |
| Winterich 2009b[^77^](#_ENREF_77) | 64 | 40-64 | African American and white | ⦁ | ⦁ | Qualitative | Semi structured, face to face interviews | Thematic analysis | Beliefs and experiences relating to masculinity and health theory |
| Woods 2004a[^66^](#_ENREF_66)** | 22 | NS | Non-Hispanic African American | ⦁ | ⦁ | Mixed methods | Interviews and focus groups | Grounded theory and thematic analysis | Influence of culture and communication on knowledge, beliefs and practices |
| Woods 2004b[^54^](#_ENREF_54)** | 22 | NS | Non-Hispanic African American | ⦁ | ⦁ | Mixed methods | Interviews and focus groups | Grounded theory and thematic analysis | Strategies for recruiting |
| Wray 2009[^100^](#_ENREF_100) | 79 | 40-80 | African American, white | ⦁ | ⦁ | Qualitative | Semi structured, face to face interviews and focus groups | Thematic analysis | Obstacles, opportunities for improving communication |
| Zimmerman 1997[^55^](#_ENREF_55) | 51 | 35-78 | Hispanic | ⦁ | ⦁ | Qualitative | Face to face interviews | Not stated | Factors influencing decision making |

*N = number of participants; NS = not stated or not applicable

** The same participants were enrolled in Woods 2004a and Woods 2004b; McFall 2003 and McFall 2006
